# Association between IFN-*γ* +874A/T and IFN-*γ*R1 (-611A/G, +189T/G, and +95C/T) Gene Polymorphisms and Chronic Periodontitis in a Sample of Iranian Population

**DOI:** 10.1155/2015/375359

**Published:** 2016-01-03

**Authors:** Zahra Heidari, Hamidreza Mahmoudzadeh-Sagheb, Mohammad Hashemi, Somayeh Ansarimoghaddam, Bita Moudi, Nadia Sheibak

**Affiliations:** ^1^Infectious Diseases and Tropical Medicine Research Center, Zahedan University of Medical Sciences, Zahedan 98167-43175, Iran; ^2^Department of Histology, School of Medicine, Zahedan University of Medical Sciences, Zahedan 98167-43175, Iran; ^3^Cellular and Molecular Research Center, Zahedan University of Medical Sciences, Zahedan 98167-43175, Iran; ^4^Department of Clinical Biochemistry, School of Medicine, Zahedan University of Medical Sciences and Health Services, Zahedan 98167-43175, Iran; ^5^Department of Periodontology, School of Dentistry, Zahedan University of Medical Sciences, Zahedan 98167-43175, Iran

## Abstract

*Background*. Interferon gamma (IFN-*γ*) is an immune regulatory cytokine that acts through its receptor and plays important role in progression of inflammatory disease such as chronic periodontitis (CP). The purpose of this study was to determine the differences in the distribution of IFN-*γ* (+874A/T) and IFN-*γ*R1 (-611A/G, +189T/G, and +95C/T) gene polymorphisms among CP and healthy individuals and to investigate relationships between these polymorphisms and susceptibility to CP.* Materials and Methods*. 310 individuals were enrolled in the study including 210 CP patients and 100 healthy controls. Single nucleotide polymorphisms at IFN-*γ* (+874A/T) and IFN-*γ*R1 (-611A/G, +189T/G, and +95C/T) were analyzed by ARMS-PCR and PCR-RFLP methods.* Results*. The significant difference was found in genotype and allele frequency of IFN-*γ* (+874A/T) gene polymorphism in chronic periodontitis patients and healthy controls. The distribution of genotypes and allele frequencies for IFN-*γ*R1 (-611A/G, +189T/G, and +95C/T) were similar among the groups and no differences in the frequencies of alleles or genotypes of IFN-*γ*R1 genetic polymorphisms variants between case and control groups were detected.* Conclusion.* The finding of this study showed that IFN-*γ* +874A/T gene polymorphism may affect susceptibility to CP, whereas IFN-*γ*R1 genetic polymorphisms at -611A/G, +189T/G, and +95C/T were not associated with this disease.

## 1. Introduction

Chronic periodontitis (CP) is a multifactorial inflammatory disease that is initiated by the accumulation of dental plaque and destroys the dental attachment apparatus. The interaction of this bacterial biofilm with the host immune system induces inflammation and immune responses, which lead to progressive attachment loss, bone loss, and eventually tooth loss. This common complex infectious disease affects 10–15 percent of the adult population [1–4].

The host inflammatory immune reaction, genetic factors, and environmental factors affect the risk of developing periodontitis. Recent studies have demonstrated that elevated levels of inflammatory biomarkers and genetic variants of some cytokines could cause susceptibility to periodontitis [4–7].

Increase of cytokines such as interferons (IFNs) has been found in infected human periodontium and their levels can be associated with progression of lesions and severity of inflammatory diseases. Interferons are a large family of cytokines that acts against viruses, tumors, and cell proliferation and acts as immunomodulatory factors [8–10].

Type II IFN (interferon-gamma) that is encoded by the interferon-gamma (IFN-*γ*) gene has stronger imunomodulatory effects than type I IFN (interferon-alpha) and plays important roles in periodontal tissue destruction [10].

IFN-*γ* also activates macrophages and is a regulatory cytokine in the process of immune reaction. Although interferon-*α* and interferon-*β* can be secreted by all cells, IFN-*γ* is expressed by CD4+ Th1 cells, cytotoxic CD8 cells, activated NK cells, mononuclear cells, and dendritic cells found in periodontal tissues. The molecular signaling pathways that lead to chronic elevation of IFN-*γ* expression in periodontal diseases are still the subject of investigation [8, 9].

Binding of IFN-*γ* to its heterodimeric receptor complex induces cellular activation and upregulation of specific genes [11–13].

This complex consists of two or more subunits IFN-*γ*R1 (ligand-binding subunit) and IFN-*γ*R2 (transmembrane accessory factor) that are encoded by chromosome 21. It is believed that both parts of the receptor complex are necessary for normal signaling. Dimerization of IFN-*γ*R1 is facilitated by binding of homodimeric IFN-*γ* with association of IFN-*γ*R2. Based on the evidence interaction of IFN-*γ* with both subunits of the receptor causes association of these parts. Cellular activation of IFN-*γ* may be induced in lower levels through an additional receptor, which is still unknown [12, 13].

The place of IFN-*γ*R expression is on nucleated cells such as some immune cells, endothelial cells, and fibroblasts [14].

The producing cell type, the nature of the stimulus, and the genetic background may influence cytokine secretion levels [7, 15].

The expression of IFN-*γ* is considerable because of its elevated transcriptional and translational expression in inflamed gingival tissues and gingival crevicular fluid (GCF). Reports have demonstrated that genetic factors may play an important role in the risk of periodontal diseases [7, 9, 16].

Focus of most genetic research in periodontitis is on gene polymorphisms. Polymorphisms, probably occurring at regulatory regions of cytokine genes, may increase susceptibility to some infectious diseases and influence the course and prognosis of the disease [7, 17].

The IFN-*γ* gene is located on chromosome 12q24 and contains four exons [8, 18].

There is a single nucleotide polymorphism +874A/T that is located at the 5′-end of a CA repeat in the first intron of the human IFN-*γ* gene. The +874 T allele is connected to the 12 CA repeats, whereas the A allele is linked with the non-12 CA repeats. The +874 T allele is associated with high IFN-*γ* expression in opposition to low expression of the A allele [8].

IFN-*γ*R1 gene is located on chromosome 6p23.3 with seven exons [14, 18, 19].

The minimal promoter region of IFN-*γ*R1 is highly polymorphic [20].

There is an association between mycobacterial infections and mutations of IFN-*γ*Rs genes. It is demonstrated that the mutations prevalently occur in IFN-*γ*R1 more than IFN-*γ*R2. Total lack of IFN-*γ*R1 expression on the cell surface results in recessive IFN-*γ*R1 defect or loss of IFN-*γ* binding completely [12].

As a result, the level of IFN-*γ*R1 expression is probably the main factor of IFN-*γ* responsiveness [20].

Polymorphisms in the genes that encode involving enzymes in the biotransformation of carcinogens have been related to cancer development [19].

Published reports have focused on the correlation of IFN-*γ*R1 promoter polymorphism and susceptibility to infectious disease [20].

Effects of IFN-*γ*R1 -611 (rs1327474) single nucleotide polymorphisms (SNPs) on the promoter activity are stronger than SNP at position -56 of IFN-*γ*R [13].

Rosenzweig et al. used a luciferase reporter system and found that G-611 carrier constructs are stronger in promoter activity than constructs carrying -611A [20].

IFN-*γ*R1 (+95C/T) SNP (rs7749390) seems to control the intron-exon splicing process and is mapped on the exon/intron splice site [18].

Similar studies on periodontal disease mainly have focused on the potentially functional polymorphisms of IFN-*γ* like +874A/T [8].

Considering the critical role of IFN-*γ* and IFN-*γ*R1 in immunity, we performed a population-based case-control study to explore the effects of genetic polymorphisms of IFN-*γ* and IFN-*γ*R1 in the risk of CP.

In present study, we examined the effects of IFN-*γ* (+874A/T) and IFN-*γ*R1 (-611A/G, +189T/G, and +95C/T) gene polymorphisms on prevalence of chronic periodontitis.

## 2. Materials and Methods

### 2.1. Subject Population

This case-control study was done on 210 CP patients and 100 healthy individuals. All subjects were exclusively Iranian ethnicities from the region of Sistan and Baluchistan.

Patients with chronic periodontitis were examined at the Periodontology Department, Dentistry Clinic of Zahedan University of Medical Sciences (ZUMS). The average age of individuals was 28.33 ± 5.765 (95 female and 115 male). All subjects had at least 20 teeth and were of good general health.

The disease diagnosis was based on physical examination, medical and dental history, probing depth (measured as the distance from the gingival margin to the bottom of the pocket), and assessment of clinical attachment loss (as the distance from the cement-enamel junction to the bottom of the periodontal pocket). Probing was performed at six sites around each tooth using a WHO periodontal probe and recording the maximum values, tooth mobility, and radiographs. The loss of alveolar bone was determined radiographically [21, 22].

The control group consisted of 100 unrelated healthy individuals who had no clinical history of periodontal disease. Controls were selected from subjects referred to the Dentistry Clinic for reasons other than periodontal disease and were matched for age, ethnicity, and gender (29.22 ± 3.597, 52 female and 48 male).

### 2.2. Exclusion Criteria

Smokers, pregnant women, and persons with history of or current cardiovascular disorders, systemic disorders such as diabetes, immunodeficiency, HIV infection, hepatitis, and viral infections, long-term use of anti-inflammatory drugs, malignant diseases, chemotherapy, and orthodontic instruments were excluded from the study.

### 2.3. Sample Collection

The study was performed with the approval of the Committees for Ethics of Zahedan University of Medical sciences (number 6210). Written informed consent was obtained from all participants.

2 mL peripheral blood was collected from all participants in Na-EDTA tubes.

### 2.4. DNA Extraction and Genetic Analysis

DNA for genetic analysis was extracted from peripheral blood samples using salting out method according to Hashemi et al. [23].

The primers that were used for genotyping were listed in Table 1.

Genotyping for the +874A/T polymorphism (rs62559044) in IFN-*γ* gene was performed by using the amplification refractory mutation system-polymerase chain reaction (ARMS-PCR) as was described previously by Pravica et al. [24].

**Figure 1:**
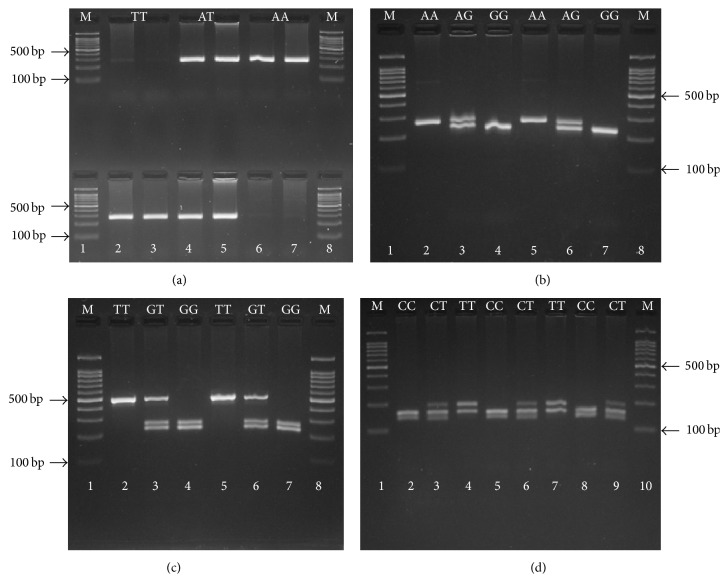
Electrophoresis pattern of ARMS-PCR for detection of IFN-*γ* (+874A/T) (a) and RFLP-PCR IFN-*γ*R1 (-611A/G), (+189T/G), and (+95C/T) gene polymorphism (resp., (b), (c), and (d)). (a) Lines 1 and 8: marker 100 bp; Lines 2 and 3: product sizes were 264 bp for forward T allele primer (FT) and reverse primer (R); Homozygote TT; Lines 6 and 7: product sizes were 264 bp for forward A allele primer (FA) and reverse primer (R); Homozygote AA; Lines 4 and 5: product sizes were 264 bp for FT, FA, and R; Heterozygote AT; (b) Lines 1 and 8: marker 100 bp; Lines 2 and 5: Homozygote AA; Lines 4 and 7: Homozygote GG; Lines 3 and 6: Heterozygote AG; (c) Lines 1 and 8: marker 100 bp; Lines 2 and 5: Homozygote TT; Lines 4 and 7: Homozygote GG; Lines 3 and 6: Heterozygote GT; (d) Lines 1 and 10: Marker 100 bp, Lines 2, 5, and 8: Homozygote CC; Lines 4 and 7: Homozygote TT; Lines 3, 6, and 9: Heterozygote CT.

PCR was performed using 2X Prime Taq Premix (Genet Bio, Korea). For each sample, we used two tubes, one for A allele and another tube for T allele. In each 0.20 mL reaction, 1 *μ*L of each primer, 1 *μ*L of genomic DNA (~100 ng/mL) and 10 *μ*L of 2X Prime Taq Premix (Genet Bio, Korea), and 7 *μ*L ddH_2_O were added. A-allele tube contained forward primer for A allele and reverse primer and T allele tube contained forward primer for T allele and reverse primer. PCR reaction was performed according to the following protocol: initial denaturation at 95°C for 5 min, 30 cycles of denaturation at 95°C for 30 s, annealing at 57.1°C for 30 s, extension at 72°C for 30 s, and final extension at 72°C for 5 min. The amplified products were separated by electrophoresis on a 2% agarose gels stained with 0.5 *μ*g/mL ethidium bromide and observed under UV light. Two sample products were available for each subject (1 for each specific A or T allele of the IFN-*γ* +874A/T variant) (Table 3, Figure 1).

(-611A/G) rs1327474, (+189T/G) rs11914, and (+95C/T) rs7749390 polymorphisms in IFN-*γ*R1 gene were genotyped using the polymerase chain reaction restriction fragment length polymorphism (PCR-RFLP) technique by specific primers and enzymes (Tables 1 and 2).

For genetic analysis of IFN-*γ*R1 (-611A/G) rs1327474 polymorphism PCR was performed using 2X Prime Taq Premix (Genet Bio, Korea). In each 0.20 mL reaction, 1 *μ*L of each primer, 1 *μ*L of genomic DNA (~100 ng/mL) and 10 *μ*L of 2X Prime Taq Premix (Genet Bio, Korea), and 7 *μ*L ddH_2_O were added. PCR reaction was performed according to the following protocol: initial denaturation at 95°C for 5 min, 30 cycles of denaturation at 95°C for 30 s, annealing at 62°C for 30 s, extension at 72°C for 30 s, and final extension at 72°C for 5 min. The PCR product (10 *μ*L) was digested using Hpy188I restriction enzyme. The G allele was digested and produced 204 bp and 29 bp fragments while the A allele was undigested and produced a 233 bp fragment. The PCR and fragments were verified on 3% agarose gels containing 0.5 *μ*g/mL ethidium bromide and were visualized under UV light (Figure 1).

Genotyping of IFN-*γ*R1 (+189T/G) rs11914 polymorphism was performed by PCR reaction containing 2X Prime Taq Premix (Genet Bio, Korea). In each 0.20 mL reaction, 1 *μ*L of each primer, 1 *μ*L of genomic DNA (~100 ng/mL) and 10 *μ*L of 2X Prime Taq Premix (Genet Bio, Korea), and 7 *μ*L ddH_2_O were added. PCR reaction was performed according to the following protocol: initial denaturation at 95°C for 5 min, 30 cycles of denaturation at 95°C for 30 s, annealing at 63.7°C for 30 s, extension at 72°C for 30 s, and final extension at 72°C for 5 min. The PCR product (10 *μ*L) was digested using TaqI restriction enzyme. The G allele was digested and produced 286 bp and 210 bp fragments while the T allele was undigested and produced a 496 bp fragment. Each reaction was verified on 2% agarose gels containing 0.5 *μ*g/mL ethidium bromide and observed under UV light (Figure 1).

PCR for genetic analysis of IFN-*γ*R1 (+95C/T) rs7749390 polymorphism was performed using 2X Prime Taq Premix (Genet Bio, Korea). In each 0.20 mL reaction, 1 *μ*L of each primer, 1 *μ*L of genomic DNA (~100 ng/mL) and 10 *μ*L of 2X Prime Taq Premix (Genet Bio, Korea), and 7 *μ*L ddH_2_O were added. PCR reaction was performed according to the following protocol: initial denaturation at 95°C for 5 min, 30 cycles of denaturation at 95°C for 30 s, annealing at 62°C for 30 s, extension at 72°C for 30 s, and final extension at 72°C for 5 min. The PCR product (10 *μ*L) was digested using BstC8I restriction enzyme. The G allele was digested and produced 87 bp, 121 bp, and 158 bp fragments and the T allele produced 208 bp and 158 fragment. The PCR and fragments were verified on 3% agarose gels containing 0.5 *μ*g/mL ethidium bromide and were observed under UV light (Figure 1).

### 2.5. Statistical Analysis

The significance of the differences in the observed frequencies of IFN-*γ* and IFN-*γ*R1 polymorphisms in the control and patient groups was computed by Chi-square test with software SPSS ver.20.0. Only the values of *P* less than 0.05 were considered significant. The risk associated with individual alleles or genotypes was as calculated by performing a multiple logistic regression analysis to estimate the odds ratio (OR) and confidence interval 95% (CI).

## 3. Results 

The demographic and clinical parameters of the CP patients and healthy controls were shown in Table 4. The CP group exhibited a significantly greater mean of PD (5.58 ± 0.63 mm versus 1.50 ± 0.86 mm) and CAL (5.44 ± 0.58 mm) and a higher percentage of sites with BOP (85.86 ± 3.68%) than the control group (*P* < 0.05).

The demographic data showed that the mean ages for patients with CP and healthy subjects did not differ between the two groups (resp., 28.33 ± 5.765 and 29.22 ± 3.597, *P* < 0.05). There were no significant differences between subjects with periodontitis and controls regarding the ethnicity and gender (Table 4).

The genotype and allele frequencies distributions among cases and controls have been shown in Table 5.

Significant difference in the genotype and allele frequencies of the IFN-*γ* gene polymorphism at positions +874A/T between CP patients and healthy subjects was found (*P* = 0.038).

The IFN-*γ*R1 polymorphisms at positions -611A/G, +189T/G, and +95C/T were not significantly different between subjects with chronic periodontitis and controls (*P* > 0.05).

To explore the potential higher-order gene-gene interactions, we performed haplotype analysis. We found 14 haplotypes (Table 6). The findings showed that haplotypes were not significantly different between subjects with CP and controls (*P* = 0.774).

## 4. Discussion

The present study investigated the association of four gene polymorphisms with CP in a sample of Iranian population. Our study was the first one to explore the polymorphism at this locus on the susceptibility to CP.

Our findings showed that IFN-*γ*R1 (+95C/T, -611A/G, and +189T/G) polymorphisms are not associated with the risk of CP in our population, whereas we found significant association for alleles and genotypes of IFN-*γ* (+874A/T) SNP between patients with CP and controls.

On the contrary, Holla et al. [8] found no significant correlation for alleles and genotypes of IFN-*γ* +874A/T polymorphism between patients with CP and controls.

Data of Erciyas et al. [25] studies showed no associations between IFN-*γ* polymorphism (+874) and generalized aggressive periodontitis too.

Loo et al. [16] study did not find any significant difference in gene investigation (IL-1b, IL-6, IFN-*γ*, and IL-10) in the CP patients and healthy subjects.

One reason for the conflicting results is various genotype and allele frequencies between different ethnic populations that confirm heterogeneity of populations; therefore a genetic risk factor in one population may not change disease susceptibility in another population [26, 27].

Different findings probably are results of the complex nature of periodontitis, which is because of the interaction between many factors such as pathogens, host immune response, and role of environment [8].

Difference in the sample size, criteria of subject selection, and ethnic diversity that could affect genetic variations may cause different result from similar studies [28].

According to the fact that a single genetic polymorphism has weak effect on immunity reactions, interactions of other genes and environment are concerned and can possibly change observed phenotype [8, 18].

The correlation between IFN-*γ* levels and variation in experimental periodontitis is clear, but the molecular mechanisms of its connection to inflammation of periodontium have not been found [8].

Interaction between some factors such as age, sex, ethnic background, and geography can potentially change disease prevalence. In other words, these elements may lead to various effects of cytokine gene polymorphisms in different population samples [7, 26].

IFN-*γ* is an immune regulator and for its binding and signaling the IFN-*γ*R1 subunit is essential [16, 29].

According to published reports, IFN-*γ* has essential role in initiation and progression of inflammatory diseases [18, 30].

Reports have demonstrated that IFN-*γ* +874A/T polymorphism is related to different levels of this cytokine and can affect the immune response and susceptibility to inflammatory diseases [8].

The T allele of the IFN-*γ* (+874A/T) found in increased producers of this cytokine indicated that polymorphism in IFN-*γ* gene causes differences in the immunoregulatory role of its molecules [16].

Findings have indicated that the +874 IFN-*γ* polymorphism was associated with two important autoimmune processes: Systemic Lupus Erythematosus and arthritis [31].

It has been demonstrated that IFN-*γ* (+874A/T) allele can change the susceptibility to tuberculosis (TB) [27, 32].

Mutations in IFN-*γ*R1 coding gene cause excessive susceptibility to mycobacterial infection and can be disruptive [16, 29].

Correlation between two IFN-*γ*R1 promoter polymorphisms and immunity against malaria in West Africa is emerging evidence that prevalent IFN-*γ*R1 variants may affect incidence of infection in the population [29].

There is emerging evidence that genetic polymorphisms of rs1327475 (-611A/G) and rs7749390 (+95C/T) were correlated with an altered risk of tuberculosis (TB) [18].

Lee et al. [33] study suggested that IFN-*γ*R1 (83G/A) polymorphism may be associated with increased predisposition to leprosy [33].

These findings confirmed that IFN-*γ* probably is a regulatory key in immune response and inflammation process.

Any number of gene polymorphisms that have any function in IFN-*γ* dependent inflammatory responses could be essential in determining susceptibility or resistance to periodontitis [32, 34].

Complex diseases genetically have heterogeneous nature. These common diseases may be caused by several genotypes at various positions on a chromosome that lead to moderate, increased, or reduced effects [32].

There are few longitudinal studies that have focused on the relationship between IFN-*γ* genotype and periodontal disease severity, alveolar bone loss, tooth loss, and response to CP treatment. There is no longitudinal study concerning the putative role of IFN-*γ* in periodontal disease outcome. Therefore, we do not know the functional consequences of these polymorphisms in our subjects [8, 25].

Undoubtedly, further investigations about serum level of IFN-*γ*, gene expression, RNA expression, stereological analysis of tissue destruction, and immunohistochemical changes in patients with these polymorphisms are necessary for interpretation of histological changes.

Additional insight into the biology of chronic periodontitis and stratified phenotypic analysis of CP would be provided by further studies and would assist in a better comprehension of molecular impression of polymorphisms.

The limitation of this study is its relatively small sample size and lack of observance of specific bacteria. Consequently, subgroup analysis was not possible. Replications in larger populations are needed to conclusively confirm or reject our findings. Subject selection with high accuracy and exact matching between groups were strengths of our study.

## 5. Conclusion

Taken together, our results suggest that IFN-*γ* +874A/T genetic polymorphism has association with susceptibility to CP in Iranian population, but IFN-*γ*R1 gene (in -611A/G, +189T/G, and +95C/T positions) is not associated with the risk of CP in this population. Larger studies are needed to confirm these findings on the relationships of genetic variations to the pathogenesis of CP.

## Figures and Tables

**Table 1:** The primers sequences used for detection of IFN-*γ* (+874A/T), IFN-*γ*R1 (-611A/G), (+189T/G), and (+95C/T) gene polymorphisms using ARMS-PCR and RFLP-PCR.

Gene	Polymorphisms	Primers	Sequence
IFN-*γ*R1	-611A/G	Forward	AGAGCAGACCTCTTCATGAGAGGCTGTCT
	rs1327474	Reverse	ACATTTTTAGAAGAGAATGAGACTTCAAA
	+95C/T	Forward	GCCATTTGGTGGTCCATTAC
	rs7749390	Reverse	TCCAGACAGCTGGAATCAGT
	+189T/G	Forward	CTCTTTCTCCTACCCCTTGTCAT
	rs11914	Reverse	CAGCGCATAATCGTATTTAAAAGTG

IFN-*γ*	+874A/T	Forward (T allele)	TTCTTACAACACAAAATCAAATCT
	rs62559044	Forward (A allele)	TTCTTACAACACAAAATCAAATCA
		Reverse	TCAACAAAGCTGATACTCCA

**Table 2:** The restriction enzymes used for digestion of IFN-*γ*R1 (-611A/G), (+189T/G), and (+95C/T) gene polymorphisms using RFLP-PCR.

Position	Restriction enzyme	PCR product
-611	Hpy188I	G allele: 204 bp and 29 bp
		A allele: 233 bp

+189	TaqI	G allele: 286 bp and 210 bp
		T allele: 496 bp

+95	BstC8I	C allele: 87 bp, 121 bp, and 158 bp
		T allele: 208 bp and 158 bp

**Table 3:** The PCR protocols for genetic analysis of IFN-*γ* (+874A/T), IFN-*γ*R1 (-611A/G), (+189T/G), and (+95C/T) gene polymorphisms using ARMS-PCR and RFLP-PCR.

Gene	Polymorphisms	Denaturation	Annealing	Extension	Final extension
IFN-*γ*R1	-611A/G	95°C for 30 s	62°C for 30 s	72°C for 30 s	72°C for 5 min
	rs1327474				
	+95C/T	95°C for 30 s	62°C for 30 s	72°C for 30 s	72°C for 5 min
	rs7749390				
	+189T/G	95°C for 30 s	63.7°C for 30 s	72°C for 30 s	72°C for 5 min
	rs11914				

IFN-*γ*	+874A/T	95°C for 30 s	57.1°C for 30 s	72°C for 30 s	72°C for 5 min
	rs62559044				

**Table 4:** Demographic data in patients with chronic periodontitis (CP) and controls.

	Patients with CP	Controls
Age		
Mean age	28.33 ± 5.765	29.22 ± 3.597
Age range	16–42	24–37
Gender		
Female	95 (45.2%)	52 (52%)
Male	115 (54.8%)	48 (48%)
Race		
Sistani	82 (39%)	42 (42%)
Baluch	71 (33.8%)	24 (24%)
Other	57 (27.1%)	34 (34%)
Clinical parameters		
BOP index (%)	85.86 ± 3.68	—
Mean PD (mm)	5.58 ± 0.63	1.50 ± 0.86
CAL (mm)	5.44 ± 0.58	—

“—” means bleeding and attachment loss were not seen in controls.

Data are *n* (%) or mean ± SD.

**Table 5:** The genotypes and allele distribution of IFN-*γ*/IFN-*γ*R1 polymorphisms in chronic periodontitis (CP) patients and controls.

Polymorphism	CP *n* = 210	Controls *n* = 100	OR 95% (CI)	*P* value
IFN-*γ* +874A/T				
AA	71 (33.8%)	20 (20%)	2.420 (CI = 1.063–5.511)	0.035
AT	117 (55.7%)	65 (65%)	1.227 (CI = 0.596–2.529)	0.579
TT	22 (10.5%)	15 (15%)	—	Ref = 1
Alleles				
A	259 (61.7%)	105 (52.5%)	1.455 (CI = 1.036–2.045)	0.031
T	161 (38.3%)	95 (47.5%)	—	Ref = 1

IFN-*γ*R1 -611A/G				
AA	67 (31.9%)	33 (33%)	0.883 (CI = 0.452–1.726)	0.715
GA	97 (46.2%)	47 (47%)	0.923 (CI = 0.450–1.893)	0.826
GG	46 (21.9%)	20 (20%)	—	Ref = 1
Alleles				
A	231 (55%)	113 (56.5%)	0.941 (CI = 0.670–1.321)	0.725
G	189 (45%)	87 (43.5%)	—	Ref = 1

IFN-*γ*R1 +189T/G				
GG	23 (11%)	8 (8%)	1.426 (CI = 0.603–3.370)	0.419
GT	62 (29.5%)	30 (30%)	1.025 (CI = 0.602–1.744)	0.927
TT	125 (59.5%)	62 (62%)	—	Ref = 1
Alleles				
G	108 (25.7%)	46 (23%)	1.159 (CI = 0.780–1.721)	0.465
T	312 (74.3%)	154 (77%)	—	Ref = 1

IFN-*γ*R1 +95C/T				
CC	54 (25.7%)	24 (24%)	—	Ref = 1
TC	95 (45.2%)	48 (48%)	0.880 (CI = 0.486–1.592)	0.672
TT	61 (29%)	28 (28%)	0.968 (CI = 0.502–1.867)	0.923
Alleles				
C	203 (48.3%)	96 (48%)	—	Ref = 1
T	217 (51.7%)	104 (52%)	0.987 (CI = 0.704–1.382)	0.938

OR: odd ratio; CI: confidence interval.

**Table 6:** The haplotype analysis for IFN-*γ*/IFN-*γ*R1 polymorphisms.

Haplotypes	CP group	Control group	*P*
	*n* (%)	*n* (%)	
AATT	62 (29.5)	31 (31.0)	*P* = 0.774 > 0.05
AGTG	16 (7.6)	9 (9)	
AGCT	5 (2.4)	4 (4.0)	
TGCG	2 (1.0)	—	
TACT	2 (1.0)	1 (1.0)	
TACG	—	1 (1.0)	
TGTT	4 (1.9)	2 (2.0)	
AACT	29 (13.8)	15 (15.0)	
AATG	46 (21.9)	19 (19.0)	
AGTT	15 (7.1)	4 (4.0)	
AACG	11 (5.2)	2 (2.0)	
AGCG	4 (1.9)	1 (1.0)	
TATT	7 (3.3)	6 (6.0)	
TATG	7 (3.3)	5 (5.0)	
Total	210 (100.0)	100 (100.0)	

## References

[1] Heidari Z., Mahmoudzadeh-Sagheb H., Hashemi M., Rigi-Ladiz M. A. (2014). Quantitative analysis of interdental gingiva in patients with chronic periodontitis and transforming growth factor-*β*1 29C/T gene polymorphisms. *Journal of Periodontology*.

[2] Armingohar Z., Jørgensen J. J., Kristoffersen A. K., Schenck K., Dembic Z. (2014). Polymorphisms in the interleukin-1 gene locus and chronic periodontitis in patients with atherosclerotic and aortic aneurysmal vascular diseases. *Scandinavian Journal of Immunology*.

[3] Preethi P. L., Rao S. R., Madapusi B. T., Narasimhan M. (2014). Immunolocalization of Ki-67 in different periodontal conditions. *Journal of Indian Society of Periodontology*.

[4] Heidari Z. (2014). The association between proinflammatory gene polymorphisms and level of gingival tissue degradation in chronic periodontitis. *Gene, Cell and Tissue*.

[5] Mesa F., O'Valle F., Rizzo M. (2014). Association between COX-2 rs 6681231 genotype and Interleukin-6 in periodontal connective tissue. A pilot study. *PLoS ONE*.

[6] Ribeiro Souto G., Queiroz C. M., de Abreu M. H. N. G., Oliveira Costa F., Alves Mesquita R. (2014). Pro-inflammatory, Th1, Th2, Th17 cytokines and dendritic cells: a cross-sectional study in chronic periodontitis. *PLoS ONE*.

[7] Heidari Z., Mahmoudzadeh-Sagheb H., Rigi-Ladiz M. A., Taheri M., Moazenni-Roodi A., Hashemi M. (2013). Association of TGF-*β*1 − 509 C/T, 29 C/T and 788 C/T gene polymorphisms with chronic periodontitis: a case-control study. *Gene*.

[8] Holla L. I., Hrdlickova B., Linhartova P., Fassmann A. (2011). Interferon-*γ* +874A/T polymorphism in relation to generalized chronic periodontitis and the presence of periodontopathic bacteria. *Archives of Oral Biology*.

[9] Zhang S., Crivello A., Offenbacher S., Moretti A., Paquette D. W., Barros S. P. (2010). Interferon-gamma promoter hypomethylation and increased expression in chronic periodontitis. *Journal of Clinical Periodontology*.

[10] Tanaka M. H., Giro E. M. A., Cavalcante L. B. (2012). Expression of interferon-*γ*, interferon-*α* and related genes in individuals with Down syndrome and periodontitis. *Cytokine*.

[11] Newport M. J., Huxley C. M., Huston S. (1996). A mutation in the interferon-*γ*-receptor gene and susceptibility to mycobacterial infection. *The New England Journal of Medicine*.

[12] Rosenzweig S. D., Holland S. M. (2005). Defects in the interferon-*γ* and interleukin-12 pathways. *Immunological Reviews*.

[13] Xiang L., Elci O. U., Rehm K. E., Marshall G. D. (2014). Associations between cytokine receptor polymorphisms and variability in laboratory immune parameters in normal humans. *Human Immunology*.

[14] Jüliger S., Bongartz M., Luty A. J. F., Kremsner P. G., Kun J. F. J. (2003). Functional analysis of a promoter variant of the gene encoding the interferon-gamma receptor chain I. *Immunogenetics*.

[15] Moreira P. R., Lima P. M. A., Sathler K. O. B. (2007). Interleukin-6 expression and gene polymorphism are associated with severity of periodontal disease in a sample of Brazilian individuals. *Clinical & Experimental Immunology*.

[16] Loo W. T. Y., Fan C.-B., Bai L.-J. (2012). Gene polymorphism and protein of human pro- and anti-inflammatory cytokines in Chinese healthy subjects and chronic periodontitis patients. *Journal of Translational Medicine*.

[17] Ebadian A. R., Radvar M., Afshari J. T. (2013). Gene polymorphisms of TNF-*α* and IL-1*β* are not associated with generalized aggressive periodontitis in an Iranian subpopulation. *Iranian Journal of Allergy, Asthma and Immunology*.

[18] Lü J., Pan H., Chen Y. (2014). Genetic polymorphisms of IFNG and IFNGR1 in association with the risk of pulmonary tuberculosis. *Gene*.

[19] de Araújo R. M. S., de Melo C. F. V., Neto F. M. (2014). Association study of SNPs of genes IFNGR1 (rs137854905), GSTT1 (rs71748309), and GSTP1 (rs1695) in gastric cancer development in samples of patient in the northern and northeastern Brazil. *Tumor Biology*.

[20] Rosenzweig S. D., Schäffer A. A., Ding L. (2004). Interferon-*γ* receptor 1 promoter polymorphisms: population distribution and functional implications. *Clinical Immunology*.

[21] Armitage G. C. (2004). Periodontal diagnoses and classification of periodontal diseases. *Periodontology*.

[22] Armitage G. C. (1999). Development of a classification system for periodontal diseases and conditions. *Annals of Periodontology*.

[23] Hashemi M., Moazeni-Roodi A. K., Fazaeli A. (2010). Lack of association between paraoxonase-1 Q192R polymorphism and rheumatoid arthritis in southeast Iran. *Genetics and Molecular Research*.

[24] Pravica V., Perrey C., Stevens A., Lee J.-H., Hutchinson I. V. (2000). A single nucleotide polymorphism in the first intron of the human IFN-*γ* gene: absolute correlation with a polymorphic CA microsatellite marker of high IFN-*γ* production. *Human Immunology*.

[25] Erciyas K., Pehlivan S., Sever T., Igci M., Arslan A., Orbak R. (2010). Association between TNF-*α*, TGF-*β*i, IL-10, IL-6 and IFN-*γ* gene polymorphisms and generalized aggressive periodontitis. *Clinical & Investigative Medicine*.

[26] Laine M. L., Crielaard W., Loos B. G. (2012). Genetic susceptibility to periodontitis. *Periodontology 2000*.

[27] Wang J., Tang S., Shen H. (2010). Association of genetic polymorphisms in the IL12-IFNG pathway with susceptibility to and prognosis of pulmonary tuberculosis in a Chinese population. *European Journal of Clinical Microbiology and Infectious Diseases*.

[28] Yücel Ö. Ö., Berker E., Mesci L., Eratalay K., Tepe E., Tezcan İ. (2015). Analysis of TNF-*α* (-308) polymorphism and gingival crevicular fluid TNF-*α* levels in aggressive and chronic periodontitis: a preliminary report. *Cytokine*.

[29] Koch O., Kwiatkowski D. P., Udalova I. A. (2006). Context-specific functional effects of IFNGR1 promoter polymorphism. *Human Molecular Genetics*.

[30] Niedzielska I., Cierpka S. (2010). Interferon *γ* in the etiology of atherosclerosis and periodontitis. *Thrombosis Research*.

[31] Bidwell J. L., Wood N. A. P., Morse H. R., Olomolaiye O. O., Keen L. J., Laundy G. J. (1999). Human cytokine gene nucleotide sequence alignments. *European Journal of Immunogenetics*.

[32] Pacheco A. G., Cardoso C. C., Moraes M. O. (2008). *IFNG* + 874T/A, *IL10* -1082G/A and *TNF*-308G/A polymorphisms in association with tuberculosis susceptibility: a meta-analysis study. *Human Genetics*.

[33] Lee S.-B., Kim B. C., Jin S. H. (2003). Missense mutations of the interleukin-12 receptor beta 1(*IL12RB1*) and interferon-gamma receptor 1 (*IFNGR1*) genes are not associated with susceptibility to lepromatous leprosy in Korea. *Immunogenetics*.

[34] Fraser D. A., Loos B. G., Boman U. (2003). Polymorphisms in an interferon-*γ* receptor-1 gene marker and susceptibility to periodontitis. *Acta Odontologica Scandinavica*.

